# Epidemiology and detection as options for control of viral and parasitic foodborne disease.

**DOI:** 10.3201/eid0304.970418

**Published:** 1997

**Authors:** L A Jaykus

**Affiliations:** Department of Food Science, North Carolina State University 27695, USA. leeann_jaykus@ncsu.edu

## Abstract

Human enteric viruses and protozoal parasites are important causes of emerging food and waterborne disease. Epidemiologic investigation and detection of the agents in clinical, food, and water specimens, which are traditionally used to establish the cause of disease outbreaks, are either cumbersome, expensive, and frequently unavailable or unattempted for the important food and waterborne enteric viruses and protozoa. However, the recent introduction of regulatory testing mandates, alternative testing strategies, and increased epidemiologic surveillance for food and waterborne disease should significantly improve the ability to detect and control these agents. We discuss new methods of investigating foodborne viral and parasitic disease and the future of these methods in recognizing, identifying, and controlling disease agents.


### Epidemiology and Detection as Options for Control of Viral and Parasitic Foodborne Disease

**Lee-Ann Jaykus**
North Carolina State University, Raleigh, North Carolina, USA

Human enteric viruses and protozoal parasites are important causes of emerging food and waterborne disease. Epidemiologic investigation and detection of the agents in clinical, food, and water specimens, which are traditionally used to establish the cause of disease outbreaks, are either cumbersome, expensive, and frequently unavailable or unattempted for the important food and waterborne enteric viruses and protozoa. However, the recent introduction of regulatory testing mandates, alternative testing strategies, and increased epidemiologic surveillance for food and waterborne disease should significantly improve the ability to detect and control these agents. We discuss new methods of investigating foodborne viral and parasitic disease and the future of these methods in recognizing, identifying, and controlling disease agents.

Emerging infectious diseases can be defined as infections that have newly appeared in a population or have existed but are rapidly increasing in incidence or geographic range (1). Agents for which a particular route of transmission is newly recognized and agents (previously unidentifiable) that are now known because of advances in detection methods should also be included in this definition. Advances in epidemiologic and detection methods during the last 10 to 20 years have placed food and waterborne human enteric viruses and protozoal parasites within this category.

Human enteric viruses and protozoa are parasitic agents that replicate in the intestines of infected hosts and are excreted in the feces. In general, the viruses are limited to human hosts, while the parasitic agents (in the form of cysts or oocytes) have a variety of human and nonhuman animal hosts. Both are transmitted primarily by the fecal-oral route, and as a result, the major source of contamination for foods and water is through contact with human and animal fecal pollution. This contamination may occur directly, through contaminated meat carcasses or poor personal hygiene practices of infected food handlers, or indirectly, through contact with fecally contaminated water or other cross-contamination routes. Viruses and parasites differ from foodborne bacterial pathogens in important ways. Because they are environmentally inert, they do not replicate in food, water, or environmental samples. Additionally, unlike bacterial pathogens, human enteric viruses and protozoal parasites are environmentally stable (2), are resistant to many of the traditional methods used to control bacterial pathogens (2), and have notably low infectious doses (3,4). This allows virtually any food to serve as a vehicle for transmission and enables these agents to withstand a wide variety of commonly practiced food storage and processing conditions (2,5).

Address for correspondence: Lee-Ann Jaykus, Department of Food Science, North Carolina State University, Box 7624, Raleigh, NC 27695, USA; fax: 919-515-7124; e-mail: leeann_jaykus@ncsu.edu.

### Epidemiology

### Human Enteric Viruses

Human enteric viruses are increasingly recognized as important causes of foodborne illness. A recent report issued by the Council for Agricultural Science and Technology ranked human enteric viruses as fifth and sixth among identified causes of foodborne disease in the United States (6). A review of U.S. national surveillance data for 1979 showed that 14 (44%) of 32 foodborne disease outbreaks in institutional settings were epidemiologically typical of viral gastroenteritis (7). Viral gastroenteritis was reported as the most common foodborne illness in Minnesota from 1984 to 1991, predominantly associated with poor personal hygiene of infected food handlers (8). Furthermore, recent data indicate that 10% of the 4,617 outbreaks of foodborne disease of unconfirmed etiology reported from 1973 to 1987 met at least two of the clinical criteria for outbreaks of acute viral gastroenteritis (8,9). The apparent failure to confirm a viral etiology in such outbreaks has been due largely to the lack of available tests and the reluctance of public health officials to use epidemiologic criteria in the classification of foodborne viral disease (9-11). The unavailability of food specimens and the failure to report outbreaks of mild gastrointestinal disease have also contributed to reporting difficulties. All of these factors have resulted in a drastic underestimate of the true scope and importance of foodborne viral infection (8).

The most common types of foodborne viral disease are infectious hepatitis due to hepatitis A virus and acute viral gastroenteritis associated with the Norwalk agent and other related small, round-structured gastrointestinal viruses (12). The human enteroviruses may also be transmitted by foodborne routes (13) and are the most commonly isolated agents in surveys of naturally occurring viral contamination in foods (14). Foodborne outbreaks due to small round viruses, parvoviruses, and astroviruses are occasionally reported (15). Rotaviruses, some adenoviruses, and hepatitis E virus are important causes of waterborne disease outbreaks, particularly in developing countries (12). Foodborne outbreaks associated with human enteric viruses are almost always due to the consumption of fecally contaminated raw or undercooked shellfish and ready-to-eat products contaminated by infected food handlers (12). Postrecovery and secondary transmission are a concern (16,17). Recent outbreaks are summarized in Table 1 (18-24).

### Parasitic Protozoa

Since 1981, enteric protozoa have become the leading cause of waterborne disease outbreaks for which an etiologic agent could be determined (5). A recent study reported that 21% of drinking water–associated outbreaks between 1991 and 1992 were attributable to parasitic agents (25). Furthermore, these agents are frequent contaminants of potable water supplies (26,27). The potential for transmission of these agents by foodborne routes is increasingly recognized (28). For instance, from 1988 to 1992, seven food-associated outbreaks of giardiasis, comprising 184 cases, were reported in the United States (29). However, since foodborne transmission is only recently documented and more than half of all reported foodborne disease outbreaks have undetermined etiology, the true importance of foodborne transmission of parasitic protozoa is unknown.

Table 1. Recent outbreaks of foodborne viral disease

| Agent | Location | Date | No. Cases | Food | Confirmation[a] | Ref. |
|---|---|---|---|---|---|---|
| HAV | Shanghai, China | Jan. 1988 | 300,000 (4% total population) | Raw clams | Yes (IEM, Hybridization, Cell culture, Experimental infection) | 18 |
| HAV | U.S. (AL, GA, FL, TN, HI) | July-Aug. 1988 | 61 | Raw oysters | Yes (Antibody capture-RT-PCR) | 19 |
| SRSV | U.S. (LA) | Nov. 1993 | 40 | Raw oysters | No | 21 |
| SRSV | U.S. (LA, MD, MS, FL, NC) | Nov. 1993 | 180 | Raw/steamed oysters | No | 22 |
| SRSV | U.S. (GA) | Dec. 1994 | 34 clusters | Steamed/ roasted oysters | No | 22 |
| SRSV | U.S. (FL, TX) | Jan. 1995 | 3 | Oysters | No | 20 |
| Norwalk | U.S. (DE) | Sept. 1987 | 191 | Commercial ice | No | 23 |
| Norwalk | U.S. (CO) | July, 1988 | 1440 | Celery/ chicken salad | No | 24 |

[a]Confimation of the virus, viral antigen, or viral nucleic acid in food specimens
HAV = hepatitis A virus; IEM = immune electron microscopy; RT-PCR = reverse transcriptase polymerase chain reaction; SRSV = small round-structured gastrointestinal virus.

The most common human enteric parasitic infections in the United States are caused by *Cryptosporidium parvum* and *Giardia lamblia*. *Cyclospora* is also an emerging enteric protozoon that has recently been associated with the consumption of contaminated fruit (30). Large communitywide waterborne outbreaks of parasitic protozoa are usually associated with surface water supplies that are either unfiltered or subjected to inadequate flocculation and filtration (5). Two large waterborne outbreaks have occurred in the United States within the last 10 years (31,32); one of these was the largest recorded waterborne disease outbreak in U.S. history (32).

### Limitations

Most of the information about viral and parasitic food and waterborne disease comes from outbreak investigations by state and local health departments and surveillance programs directed by the Centers for Disease Control and Prevention (CDC). However, since many of these diseases are not reportable and surveillance is based on voluntary reporting by state health departments, the magnitude of this disease problem is underestimated. This is exacerbated by a reluctance to use epidemiologic criteria in the classification of foodborne viral disease and the failure to report mild outbreaks of gastrointestinal disease. Furthermore, since investigation generally follows an outbreak, important information and samples may have been destroyed, consumed, or lost to inaccurate recall. Since many of these outbreaks are small and confined, epidemiologic investigation may be limited by the resources available to state and local health departments. Difficulties in investigating and reporting are further complicated by the fact that the enteric protozoa cause common opportunistic infections in the immunocompromised, and the role of foods in these diseases has not been studied. Likewise, the role of the foodborne transmission route in sporadic disease and the importance of carrier states and secondary illness after a primary foodborne disease outbreak are poorly characterized.

### Detection

### Clinical Samples

Illness caused by human enteric viruses can be suspected epidemiologically by considering incubation period and illness duration analysis, classic viral gastroenteritis symptoms, and the absence of bacterial or parasitic pathogens in stool samples (10,11). Laboratory confirmation of human enteric viral infection has been based on a rise in specific antibody to the virus, or alternatively, the demonstration of virus particles, antigen, or nucleic acid in stools. The detection methods most often applied to clinical samples have included immune electron microscopy, radioimmunoassay, and enzyme immunoassay (33). The usefulness of these assays has been reduced by low detection limits ($>10^4$-$10^5$ particles/ml) and the inability to cultivate Norwalk-like viruses in vitro, which has limited the supply of viral antigen available for developing reagents (8). In addition, the Norwalk agent is only one of several small round-structured gastrointestinal viruses that cause outbreaks with similar clinical and epidemiologic features (8).

Before 1981, parasitic disease in humans was diagnosed histologically by identifying the life cycle stages of parasitic agents in the intestinal mucosa (34). More recently, clinical diagnosis has involved methods to concentrate parasitic agents from stool specimens followed by a variety of fluorescent or immunofluorescent staining techniques and subsequent microscopic examination (34). Serodiagnostic methods have been developed (35,36), but additional evaluations are needed to confirm the diagnostic utility of these methods.

### Environmental, Food, and Water Samples

Failure to confirm a viral and parasitic etiology in foodborne outbreaks has also been due to the lack of adequate methods to detect the causative agents in environmental samples. Like bacterial pathogens in food and water, viruses and parasites are frequently present in small numbers. However, unlike traditional food microbiologic techniques, which have relied on cultural enrichment and selective plating to increase cell numbers and differentiate pathogens in background microflora, techniques to detect human enteric viruses and parasitic protozoa require live mammalian cells for growth. For this reason, standard methods to detect enteric bacteria in foods cannot be used; instead, detection requires an initial concentration step, often from large volumes of food or water, followed by mammalian cell culture infectivity assay or immunofluorescent staining.

Concentration methods are usually cumbersome, and yields are less than optimal. Both cell culture infectivity and immunofluorescent staining are expensive and slow and require highly trained personnel. Furthermore, mammalian cell culture lines are largely unavailable for the epidemiologically important foodborne viruses. Alternative detection methods based on immunologic and molecular methods have been reported; recent methodologic developments have focused on overcoming barriers to detection, such as improving recovery efficiencies and detection limits and preventing inhibitions due to food-related compounds.

### Human Enteric Viruses in Foods

### Concentration

Two general schemes for the concentration of human enteric viruses from foods have been reported: extraction-concentration methods and adsorption-elution-concentration methods (Figure). The general purpose of concentration is to provide a high recovery of infectious virus in a low-volume aqueous solution free of cytotoxic materials. Both schemes employ conditions favoring the separation of viruses from shellfish tissues (most of the developmental work has used shellfish as model food commodities), primarily through the use of filtration, precipitation, polyelectrolyte flocculation, and solvent extraction. While either method can be used, adsorption-elution-precipitation methods have been favored in recent years (2,37). Virus yields after concentrations are 10% to 90% (38).

### Detection

Traditional methods to directly detect viruses in foods after concentration have been based on the ability of enteric viruses to infect live mammalian cells in culture. Quantal and enumerative methods using a variety of mammalian cell culture lines, generally from primate kidneys, have been reported. Such approaches have been limited because levels of contaminating virus generally are low (1-200 infectious units per 100 grams of shellfish) (39), residual food components interfere with assays (38), and the epidemiologically important viruses do not replicate (small round-structured gastrointestinal viruses) or replicate poorly (hepatitis A virus) in mammalian cell culture (2). Alternative methods such as enzyme-linked

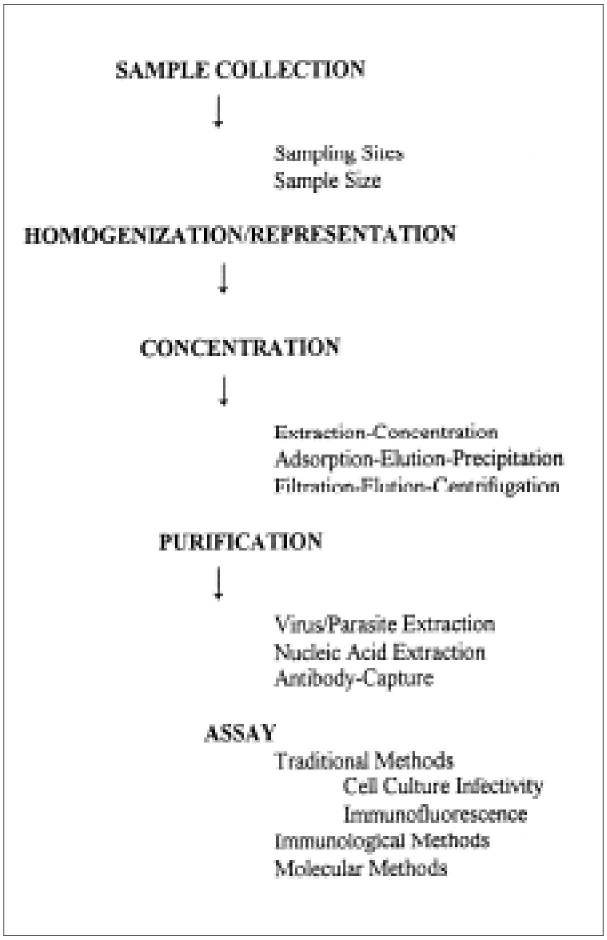

Figure. General steps in the isolation of human enteric viruses and parasitic protozoa from foods.

immunosorbent assay (ELISA) and DNA/RNA probes have been reported but are limited by high detection limits (>$10^3$ infectious units), unavailability of reagents, and poor sample quality (2). These difficulties are illustrated by the confirmation of viral contamination in a food in only two reported instances (18,19).

The in vitro enzymatic amplification method of polymerase chain reaction (PCR) offers an opportunity to enrich a single specific nucleic acid sequence up to a millionfold and hence provides a sensitive and specific method with a theoretical detection limit of one virus unit. This method is readily adaptable to the detection of RNA viruses by preceding the PCR with a brief reverse transcription (RT) step, hence the designation RT-PCR. The recent cloning of the Norwalk agent and related small round-structured gastrointestinal viruses has provided an opportunity to develop effective molecular detection methods for these previously nondetectable agents (40,41).

The application of PCR methods to the detection of human enteric viruses in foods is an area of active research. However, the development of such methods is complicated by low levels of contamination, high sample volumes, and the presence of food components, which may interfere with enzymatic amplification reactions. To address these issues, three alternative approaches have been used to simultaneously reduce sample volumes and the level of interfering compounds. The most frequently applied approach involves isolating and purifying nucleic acids (RNA) from the food sample before RT-PCR (42-47). A second approach combines capture of the virus with specific antibody followed by nucleic acid amplification by using RT-PCR (19,48). In the third approach, the intact virus particle is concentrated and purified from the complex food matrix resulting in sample volume reduction and removal of inhibitors, followed by subsequent heat release of viral nucleic acid from the virion capsid and RT-PCR (49-51). All three methods have been applied to various shellfish species, and in some cases, to other food commodities and naturally contaminated field shellfish specimens. The methods are summarized in Table 2, along with optimized virus detection levels (19,42-51). A combined approach was reported by Chung et al. (51), who successfully detected human enteroviruses and hepatitis A virus in naturally contaminated oyster samples after viral amplification in mammalian cell cultures. Despite enormous strides in the ability to detect human enteric viruses with PCR, the technique is still limited by the absence of effective concentration methods, the presence of enzymatic inhibitors, and the inability to distinguish between infectious and noninfectious virions.

Table 2. Emerging detection methods for human enteric viruses in foods

| Pathogen | Sample | Detection limit | Field app. | Ref. |
|---|---|---|---|---|
| Nucleic Acid Extraction/RT-PCR | | | | |
| HAV | Clams | 2000 particles/g (10 PFU/g) | No | 44 |
| Poliovirus | Oysters | 38 PFU/20 g (2 PFU/g) | No | 42 |
| HAV | Clams/ Oysters | 100 PFU/1.5 g (67 PFU/g) | No | 43 |
| Norwalk | Clams/ Oysters | 5-10 PCRU/1.5 g (3-7 PCRU/g) | No | 43 |
| Poliovirus | Oysters/ Mussels | 10 PFU/5 g (2 PFU/g) | No | 45 |
| SRSV | Oysters/ Mussels | Not Specified | Yes | 46 |
| Poliovirus, HAV | Clams | 100 PFU/50 g (2 PFU/g) | No | 50 |
| Norwalk | Clams | 1000 PCRU/50 g (20 PCRU/g) | No | 50 |
| Norwalk | Various | 20-200 PCRU/10 g (2-20 PCRU/g) | No | 47 |
| Antibody-Capture/RT-PCR | | | | |
| HAV | Clams/ Oysters | Not Specified | No | 48 |
| HAV | Oysters | Not Specified | Yes | 19 |
| Virion Concentration | | | | |
| Poliovirus, HAV | Oysters | 10 PFU/50 g (0.02 PFU/g) | Yes | 49, 51 |
| Norwalk | Oysters | 4500 PCRU/50 g (90 PCRU/g) | No | 49 |
| Poliovirus, HAV | Clams | 1000 PFU/50 g (20 PFU/g) | No | 50 |
| Norwalk | Clams | 100 PCRU/50 g (2 PCRU/g) | No | 50 |

HAV = hepatitis A virus; RT-PCR = reverse transcriptase polymerase chain reaction; PCRU = PCR-amplifiable units; SRSV = small round-structured gastrointestinal virus.

### Enteric Parasitic Agents

### Concentration

Like viruses, parasitic protozoa are usually present in low concentrations in contaminated water and hence must be concentrated from large volumes of water before detection (Figure). *Giardia* cysts and *Cryptosporidium* oocysts are concentrated from 100 to 1,000 liters of water by filtration through yarn-wound filters. Retained particulates are eluted from the filters and reconcentrated by centrifugation. The pelleted cysts and oocysts are then separated from particulate debris by flotation on Percoll-sucrose gradients, followed by subsequent detection. With this method, a concentrate of less than 5 ml can be provided for final detection (52). While recovery efficiencies as high as 100% have been reported (53), recovery is generally poor and greatly affected by water quality and particulate matter (53,54). Alternative methods using calcium carbonate precipitation can concentrate *Cryptosporidium* oocysts from water with concentration efficiencies as high as 63% (55).

### Detection

Traditional methods to detect parasitic agents from water sample concentrates have been based on immunofluorescent staining of filtered sample concentrates (52). The sample concentrate is filtered through cellulose acetate filters and commercial kits that use fluorescein isothiocyante–labeled monoclonal antibodies applied for immunofluorescent staining. The stained filters are examined under an ultraviolet microscope, and cysts and oocysts are classified according to immunofluorescence, size, shape, and internal morphologic characteristics. The results are reported as presumptive and confirmed cysts and oocysts per 100 liters of water (52). Confirmation is based on the ability to visualize organelles under light microscopy. This method is extremely limited because it is time-consuming and expensive, requires highly skilled personnel, and does not indicate viability of the cysts or oocysts; in addition, cross-reactions of monoclonal antibodies with algal cells and debris interfere with the interpretation of results (56).

Because of the limitations of immunofluorescence assays, alternative strategies for the detection of protozoal parasites in environmental and water samples are being sought. Most of the approaches use the traditional methods of concentration, in conjunction with alternative detection methods. In many cases, the inclusion/exclusion of fluorogenic dyes is used to enhance morphologic examination; in particular, 4,6-diamidino-2-phenyl indole and propidium iodide have been used to facilitate detection and also assess viability (57). Two recent methodologic developments include cell sorting/particle counting approaches and molecular approaches. In many cases, a combination of approaches is used. Particle-counting approaches include the Fluorescence-Activated Cell Sorting system, a laser-based particle counter that is able to simultaneously sort particles, sense fluorescence, and determine size (58). This system is being used to detect *Giardia* and *Cryptosporidium* in water samples in England and Australia (55). Differentially stained cysts and oocysts may be visualized microscopically with cooled charge couple devices (58,59). Molecular approaches that apply DNA hybridization to the detection of *Giardia* species have been reported (60). A method that combines fluorescence and in situ hybridization with confocal microscopy has been applied to both detect and speciate *Giardia* cysts (61). PCR methods have been developed to detect *Giardia* and *Cryptosporidium* (62-64). While PCR methods have the potential to detect one single infectious unit and may be applied to discriminate pathogenic from nonpathogenic species, they remain limited because of enzymatic inhibition, the inability to discriminate between viable and nonviable organisms, and the current absence of quantitative assay.

Several methods in development are combinations of multiple detection approaches. A combined method, designated the electrorotation assay, couples filtration and subsequent elution with affinity immunocapture by using paramagnetic beads. By inducing an electric field on a microscope with a special stage attachment, the organism-bead complexes rotate in a characteristic pattern that enables detection of parasitic protozoa (52). Several investigators are also working on methods that couple cell culture infectivity with immunostaining, thereby providing detection with simultaneous indication of viability (M. Sobsey, pers. comm.). Clinical ELISA kits have been evaluated for use in environmental water samples with reported detection limits of fewer than 10 cysts or oocyts (65); however, cross-reaction with algae continues to be a problem. Emerging detection approaches are summarized in Table 3 (58-64). Methods to concentrate and detect parasitic protozoa specifically from foods are under development.

### Considerations

Although prototype alternative and rapid methods to detect human enteric viruses and parasitic protozoa in foods and water have been reported, multiple barriers must be overcome before these methods are applicable to routine monitoring. To obtain sample representation and detection sensitivity adequate for the low levels of contamination found with these enteric pathogens in naturally contaminated environmental specimens, large sample volumes of food and water need to be processed. While some of the detection methods begin with large samples, many do not consider sample size, which limits the sensitivity of the assay procedure from the beginning. The approaches then applied to concentrate and purify the pathogens from the samples do not only need to be reasonably efficient but also need to produce a concentrate low in volume and free of inhibitory compounds.

Table 3. Emerging detection methods for parasitic protozoa in water

| Pathogen | Detection limit | Viability | Differentiation | Ref. |
|---|---|---|---|---|
| Flow Cytometry with Fluorescent Imaging and CCD | | | | |
| *Cryptosporidium* | NR | Yes, using differential fluorogenic vital dyes | NR | 58, 59 |
| DNA Hybridization | | | | |
| *Giardia* (16s-like rDNA) | 1 cyst | NR | No | 60 |
| *Giardia* (16s-like rDNA) | NR | NR | Yes-in situ hybridization with differential fluorescence *G. lamblia; G. muris* | 61 |
| Polymerase Chain Reaction | | | | |
| *Giardia* (Giardin gene) | 1 cyst | Partial, using mRNA as target; Depends on inactivation method | NR | 62 |
| *Giardia* (Giardin gene) | 1 cyst | NR | Yes-primer sequence *G. duodenalis* | 63 |
| *Cryptosporidium* (Target not specified) | 20 oocysts | No | Yes-by hybridization *C. parvum* | 64 |

CCD = cooled charge couple device; NR = not reported

To complicate matters further, different food commodities have differing types and levels of inhibitors, many of which have remained recalcitrant to almost all removal processes.

The relationship between detection by molecular and immunologic approaches and the subsequent viability or infectivity of these enteric pathogens remains a concern. While investigators have addressed these issues using in vitro excystation, inclusion and exclusion of vital dyes, animal infectivity (parasitic protozoa) (57,66,67), and assays combining mammalian cell culture infectivity with alternate detection strategies (viruses) (51; M. Sobsey, pers. comm.), these methods are not useful for the nonculturable human enteric viruses; they are expensive, time-consuming, and not readily amenable to routine diagnostic work. Establishing quantitative detection methods also needs further research.

The development of detection methods is also limited by the current state of knowledge. For instance, while recent sequencing evidence indicates that the Norwalk-like small round-structured gastrointestinal virus group consists of multiple members having the same physical and genomic characteristics as other viruses in the family Caliciviridae (40,41), considerable sequence and antigenic diversity remain among the members of this group (68-70). While PCR primers for these genetically diverse agents have been reported (46,71), development of universal detection methods is clearly limited until more complete information about this group of human enteric viruses is available.

Research is needed to develop and refine the prototype protocols into collaboratively tested methods that could be routinely and expeditiously used to evaluate the microbiologic safety of food products. In general, future research needs for the routine application of alternative methods to detect enteric viral and parasitic protozoal contamination in foods requires development of the following: 1) simple, rapid, and cost-effective extraction and concentration procedures; 2) simple and reliable methods for the removal of inhibitors; 3) methods that are not restricted by food product; and 4) quantitative approaches for assessing the relative levels of contamination.

### Conclusions

Improved epidemiologic surveillance, predominantly through the creation of population-based centers that will focus on the epidemiology and prevention of food and waterborne infectious disease (72), should improve knowledge regarding viral etiology in foodborne disease outbreaks and the clinical and economic importance of viral and parasitic disease agents. Data obtained from these studies may elucidate the role of foods in sporadic disease as well as in secondary spread.

Current research programs under way at CDC, U.S. Department of Agriculture, and Food and Drug Administration, as well as extramural funding through the National Institutes of Health and U.S. Department of Agriculture programs, should promote the development, testing, and dissemination of rapid and accurate detection methods for viral and parasitic foodborne disease agents. Such research will continue to be important as testing regulations, such as the landmark Environmental Protection Agency Information Collections Requirement Rule, and the new U.S. Department of Agriculture Pathogen Reduction: Hazard Analysis and Critical Control Points (HACCP) Systems Rule, emerge in the future. From a clinical standpoint, the development of inexpensive and widely available reagents has been improved through recent developments in molecular biology (33). This will increase the number of facilities able to perform diagnostic testing, which will ultimately facilitate epidemiologic investigation. From a food safety standpoint, improved detection methods should eventually provide a regulatory option for monitoring food safety, particularly in the economically important shellfish species and in detecting viral contamination due to human handling, or parasitic protozoal contamination from animal wastes. The development of rapid detection methods will also aid in the evaluation of control strategies for viral and protozoal contamination of foods. For instance, depuration, thermal processing, and irradiation for the control of foodborne viral contamination in shellfish need to be further evaluated. In the case of contamination by infected food handlers and animal wastes, the availability of rapid methods will increase our understanding of this transmission route and help determine critical control points for HACCP approaches to control the transmission of these foodborne disease agents. This will allow the integration of a true farm-to-table food safety approach for the control of viral and parasitic food and waterborne disease.

In the United States, there is currently a clear regulatory mandate to evaluate food safety risks within a systematic conceptual framework in the form of quantitative microbial risk assessment. The application of quantitative risk assessment to food safety issues has been hampered by the lack of available data regarding prevalence, transmission, infectious dose, and behavior of microorganisms in foods; this has been particularly true for emerging pathogens. Improved epidemiologic and detection methods should dramatically affect the ability to evaluate food safety risks through risk assessment strategies. For instance, by using emerging epidemiologic data in conjunction with epidemiologic modeling, statistical overview analysis (meta-analysis), and geographic information systems, scientists should be able to improve hazard analysis and exposure assessment and provide a clearer picture of disease transmission patterns. More rapid, accurate, and readily available detection methods should allow determination of prevalence of contamination and disease, and when quantitative, should provide a means of assessing dose-response relationships. Together these will improve subsequent risk characterization, allowing regulators to quantitate the magnitude of the problem, evaluate risk reduction strategies, and prioritize competing risks.

The combination of increased surveillance, improved detection methods, and testing requirements should result in a marked improvement in the ability to detect, investigate, and control food and waterborne enteric viral and parasitic protozoal agents. Taken together, these approaches promise to provide increased information necessary to assess risks, control disease, and ultimately improve public health in the next century.

### References

1. Morse SS. Factors in the emergence of infectious disease. Emerg Infect Dis 1995;1:5-10.
2. Jaykus LA, Hemard MT, Sobsey MD. Human enteric pathogenic viruses. In: Hackney CR, Peirson MD, editors. Environmental indicators and shellfish safety. New York: Chapman and Hall; 1994.
3. DuPont HL, Chappell CL, Sterling CR, Okhuysen PC, Rose JB, Jakubowski W. The infectivity of *Cryptosporidium parvum* in healthy volunteers. N Engl J Med 1995;332:855-9.
4. Haas CN. Estimation of risk due to low doses of microorganisms: a comparison of alternative methodologies. Am J Epidemiol 1983;118:573-82.
5. Moe CL. Waterborne transmission of infectious agents. In: Hurst CJ, Knudsen GR, McInerney MJ, Stenzenbach LD, Walter MV, editors. Manual of environmental microbiology. Washington (DC): American Society for Microbiology; 1996.

6. Council for Agricultural Science and Technology. Foodborne pathogens: risks and consequences. Washington (DC): Library of Congress No. 122; 1994.
7. Jenkins S, Horman JT, Isreal E, Cukor G, Blacklow NR. An outbreak of Norwalk-related gastroenteritis in a boys' camp. American Journal of Diseases of Children 1985;139:787-9.
8. Hedberg CW, Osterholm MT. Outbreaks of foodborne and waterborne viral gastroenteritis. Clin Microbiol Rev 1993;6:199-210.
9. Bean NH, Griffin PM. Foodborne disease outbreaks in the United States, 1973-1987: pathogens, vehicles, and trends. Journal of Food Protection 1990;53:804-17.
10. Kaplan JE, Feldman R, Campbell DS, Lookabaugh C, Gary W. The frequency of a Norwalk-like pattern of illness in outbreaks of acute gastroenteritis. Am J Public Health 1982;72:1329-32.
11. Kaplan JE, Gary GW, Baron RC, Singh N, Schonberger LB, Feldman R, Greenberg HB. Epidemiology of Norwalk gastroenteritis and the role of Norwalk virus in outbreaks of acute nonbacterial gastroenteritis. Ann Intern Med 1982;96:756-76.
12. Cliver DO, Ellender RD, Fout GS, Shields PA, Sobsey MD. Foodborne viruses. In: Vanderzant C, Splittstoesser DF, editors. Compendium of methods for the microbiological examination of foods. Washington (DC): American Public Health Association; 1992.
13. Centers for Disease Control. Foodborne and waterborne disease outbreaks annual summary 1976. Washington (DC): U.S. Department of Health, Education, and Welfare Publication No. CDC 78-8185; 1978.
14. Gerba CP, Goyal SM. Detection and occurrence of enteric viruses in shellfish: a review. Journal of Food Protection 1978;41:743-54.
15. Appleton H. Small round viruses: classification and role in food-borne infections. In: Ciba Foundation Symposium. Novel Diarrhoea Viruses. Chinchester, England: Wiley Publishers; 1987.
16. Heun EM, Vogt RL, Hudson PJ, Parren S, Gary GW. Risk factors for secondary transmission in households after a common-source outbreak of Norwalk gastroenteritis. Am J Epidemiol 1987;126:1181-6.
17. White KE, Osterholm MT, Mariotti JA, Korlath JA, Lawrence DH, Ristinen TL, Greenberg HB. A foodborne outbreak of Norwalk virus gastroenteritis: evidence for post-recovery transmission. Am J Epidemiol 1986;124:120-6.
18. Xu ZY, Li ZH, Wang JX, Xiao ZP, Dong DH. Ecology and prevention of a shellfish-associated hepatitis A epidemic in Shanghai, China. Vaccine 1992;(Suppl 1):S67-8.
19. Desenclos JCA, Klontz KC, Wilder MH, Nainan OV, Margolis HS, Gunn RA. A multistate outbreak of hepatitis A caused by the consumption of raw oysters. Am J Pub Health 1991;81:1268-72.
20. Centers for Disease Control and Prevention. Multistate outbreak of viral gastroenteritis associated with consumption of oysters-Apalachicola Bay, Florida, December 1995-January 1995. MMWR Morb Mort Wkly Rpt 1995;44:37-9.
21. Centers for Disease Control and Prevention. Viral gastroenteritis associated with consumption of raw oysters-Florida, 1993. MMWR Morb Mort Wkly Rpt 1994;43:446-9.
22. Centers for Disease Control and Prevention. State outbreak of viral gastroenteritis related to consumption of oysters-Louisiana, Maryland, Mississippi, and North Carolina, 1993. MMWR Morb Mort Wkly Rpt 1993;42:945-8.
23. Cannon RO, Poliner RJ, Hirshhorn RB, Rodeheaver DC, Silverman PR, Brown EA, et al. A multistate outbreak of Norwalk virus gastroenteritis associated with consumption of commercial ice. J Infect Dis 1991;164:860-4.
24. Warner RD, Carr RW, McClesky FK, Johnson PC, Goldy Elmer LM, Davison VE. A large nontypical outbreak of Norwalk virus: gastroenteritis associated with exposing celery to nonpotable water and with *Citrobacter freundii*. Arch Intern Med 1991;151:2419-24.
25. Centers for Disease Control and Prevention. Surveillance for waterborne disease outbreaks-United States, 1991-1992. MMWR Morb Mort Wkly Rpt 1993;42:1-22.
26. LeChevallier MW, Norton WD, Lee RG. *Giardia* and *Cryptosporidium* spp. in filtered drinking water supples. Appl Environ Microbiol 1991;57:2617-21.
27. Rose JB, Gerba CP, Jakubowski W. Survey of potable water supplies for *Cryptosporidium* and *Giardia*. Environ Sci Technol 1991;25:1393-400.
28. Bean NH, Griffin PM, Goulding JS, Ivey CB. Foodborne disease outbreaks, 5-year summary, 1983-1987. MMWR Morb Mort Wkly Rpt 1990;39(SS-1):15-57.
29. Bean NH, Goulding JS, Lao C, Angulo FJ. Surveillance for foodborne disease outbreaks-United States, 1988-1992. MMWR Morb Mort Wkly Rpt 1996;45(SS-5):1-66.
30. Ortega YR, Sterling CR, Gilman RH, Cama VA, Diaz F. *Cyclospora* species—A new protozoan pathogen of humans. N Engl J Med 1993;328:1308-12.
31. Hayes EB, Matte TD, O'Brien TR, McKinley TW, Logsdon GS, Rose JB, et al. Large community outbreak of cryptosporidiosis due to contamination of a filtered public water supply. N Engl J Med 1989;320:1372-6.
32. MacKenzie WR, Hoxie NJ, Proctor ME, Gradus MS, Blair KA, Peterson DE, et al. A massive outbreak in Milwaukee of *Cryptosporidium* infection transmitted through the public water supply. N Engl J Med 1994;331:161-7.
33. Kapikian AJ, Estes MK, Chanock RM. Norwalk group of viruses. In: Fields BN, Knipe DM, Howley PM, editors. Fields Virology. Philadelphia: Lippencott-Raven Publishers, Inc.; 1996.
34. Current WL, Garcia LS. Cryptosporidiosis. Clin Microbiol Rev 1991;4:324-58.
35. Campbell PN, Current WL. Demonstration of serum antibodies to *Cryptosporidium* sp. in normal and immunodeficient humans with confirmed infections. J Clin Microbiol 1983;18:165-9.
36. Ungar BLP, Soave R, Fayer R, Nash TE. Enzyme immunoassay detection of immunoglobulin M and G antibodies to *Cryptosporidium* in immunocompetent and immunocompromised persons. J Infect Dis 1986;153:570-8.
37. DeLeon R, Jaykus L. Detection of bacteria and viruses in shellfish. In: Hurst CJ, Knudsen GR, McInerney MJ, Stenzenbach LD, Walter MV, editors. Manual of environmental microbiology. Washington (DC): American Society for Microbiology; 1996.

38. Sobsey MD, Carrick RJ, Jensen HR. Improved methods for detecting enteric viruses in oysters. Appl Environ Microbiol 1978;36:121-8.
39. Cole MT, Kilgen MB, Reily LR, Hackney CR. Detection of enteroviruses, bacterial indicators, and pathogens in Louisiana oysters and their overlying waters. Journal of Food Protection 1986;49:596-601.
40. Jiang X, Graham DY, Wang K, Estes MK. Norwalk virus genome cloning and characterization. Science 1990;250:1580-3.
41. Lambden PR, Caul EO, Ashley CR, Clarke IN. Sequence and genome organization of a human small round-structured (Norwalk-like) virus. Science 1993;259:516-9.
42. Atmar RL, Metcalf TG, Neill HF, Estes MK. Detection of enteric viruses in oysters by using the polymerase chain reaction. Appl Environ Microbiol 1993;59:631-5.
43. Atmar RL, Neill HF, Romalde JL, LeGuyader F, Woodley CM, Metcalf TG, Estes MK. Detection of Norwalk virus and hepatitis A virus in shellfish tissues with the PCR. Appl Environ Microbiol 1995;61:3014-8.
44. Goswami BB, Koch WH, Cebula TA. Detection of hepatitis A in *Mercenaria mercenaria* by coupled reverse transcription and polymerase chain reaction. Appl Environ Microbiol 1993;59:2765-70.
45. Lees DN, Henshilwood K, Dore WJ. Development of a method for detection of enteroviruses in shellfish by PCR with poliovirus as a model. Appl Environ Microbiol 1994;60:2999-3005.
46. Lees DN, Henshilwood K, Green J, Gallimore CI, Brown DWG. Detection of small round structured viruses in shellfish by reverse transcription-PCR. Appl Environ Microbiol 1995;61:4418-24.
47. Gouvea V, Santos N, Carmo Timenetsky M, Estes MK. Identification of Norwalk virus in artificially seeded shellfish and selected foods. J Virol Methods 1994;48:177-87.
48. Deng MY, Day SP, Cliver DO. Detection of hepatitis A virus in environmental samples by antigen-capture PCR. Appl Environ Microbiol 1994;60:1927-33.
49. Jaykus L, DeLeon R, Sobsey MD. A virion concentration method for detection of human enteric viruses in oysters by PCR and oligoprobe hybridization. Appl Environ Microbiol 1996;62:2074-80.
50. Dix A. Development of methods to extract human enteric viruses from hard-shelled clams for detection by reverse transcriptase-polymerase chain reaction (RT-PCR) and oligoprobe hybridization (OP) [thesis]. Raleigh (NC): North Carolina State University; 1997.
51. Chung H, Jaykus L, Sobsey MD. Improved detection of human enteric viruses in field oyster specimens by in vivo and in vitro amplification of nucleic acids. Appl Environ Microbiol 1996;62:3772-8.
52. Schaefer III FW. Detection of protozoan parasites in source and finished drinking waters. In: Hurst CJ, Knudsen GR, McInerney MJ, Stenzenbach LD, Walter MV, editors. Manual of Environmental Microbiology. Washington (DC): American Society for Microbiology; 1996.
53. LeChevallier MW, Norton WD, Siegel JE, Abbaszadegan M. Evaluation of the immunofluorescence procedure for detection of *Giardia* cysts and *Cryptosporidium* oocyts in water. Appl Environ Microbiol 1995;61:690-7.
54. Nieminski EC, Schaefer III FW, Ongerth JE. Comparison of two methods for the detection of *Giardia* cysts and *Cryptosporidium* oocytes in water. Appl Environ Microbiol 1995;61:1714-9.
55. Versey G, Slade JS, Byrne M, Shepard K, Fricker CR. A new method for the concentration of *Cryptosporidium* oocysts from water. J Appl Bacteriol 1993;75:82-6.
56. Rodgers MR, Flanigan DJ, Jakubowski W. Identification of algae which interfere with the detection of *Giardia* cysts and *Cryptosporidium* oocysts and a method for alleviating this interference. Appl Environ Microbiol 1995;61:3759-63.
57. Campbell AT, Robertson LJ, Smith HV. Viability of *Cryptosporidium parvum* oocytes: correlation of in vitro excystation with inclusion/exclusion of fluorogenic vital dyes. Appl Environ Microbiol 1992;58:3488-93.
58. Campbell AT, Robertson LJ, Smith HV. Novel methodology in the detection of *Cryptosporidium parvum*: a comparison of cooled charge coupled device (CCD) and flow cytometry. Water Science Technology 1993;27:89-92.
59. Campbell AT, Haggart R, Robertson LJ, Smith HV. Fluorescent imaging of *Cryptosporidium* using a cooled charge couple device (CCD). J Microbiol Methods 1992;16:169-74.
60. Abbaszadegan M, Gerba CP, Rose JB. Detection of *Giardia* cysts with a cDNA probe and applications to water samples. App Environ Microbiol 1991;57:927-31.
61. Erlandsen SL, VanKeulen H, Gurien A, Jakubowski W, Schafer III FW, Wallis P, et al. Molecular approach to speciation and detection of *Giardia*: fluorochrome rDNA probes for identification of *Giardia lamblia*, *Giardia muris*, and *Giardia ardeae* in laboratory and environmental samples by in situ hybridization. In: Thompson RCA, Reynoldson JA, Lymbery AJ, editors. Giardia: from molecules to disease. Oxford: CAB International; 1994.
62. Mahbubani MH, Bej AK, Perlin M, Schaefer III FW, Jakubowski W, Atlas RM. Detection of *Giardia* cysts by using polymerase chain reaction and distinguishing live from dead cysts. Appl Environ Microbiol 1991;57:3456-61.
63. Mahbubani MH, Bej AK, Perlin M, Schaefer III FW, Jakubowski W, Atlas RM. Differentiation of *Giardia duodenalis* and other *Giardia* spp. by using polymerase chain reaction and gene probes. J Clin Microbiol 1992;30:74-8.
64. Webster KA, Pow JDE, Giles M, Catchpole J, Woodward MJ. Detection of *Cryptosporidium parvum* using a specific polymerase chain reaction. Vet Parasitol 1993;50:35-44.
65. de la Cruz AA, Sivaganesan M. Detection of Giardia and Cryptosporidium spp. in source water samples by commercial enzyme-immunosorbant assay. In: Proceedings 1994 Water Technology Conference; 1994 Nov 6-10; San Francisco, California. Denver (CO): American Water Works Association, 1995.
66. Sauch JF, Flanigan D, Galvin ML, Berman D, Jakubowski W. Propidium iodide as an indicator of *Giardia* cyst viability. Appl Environ Microbiol 1991;57:3243-7.
67. Schupp DG, Erlandsen SL. A new method to determine *Giardia* cyst viability: correlation of florescein diacetate and propidium iodide staining with animal infectivity. Appl Environ Microbiol 1987;53:704-7.

68. Moe CL, Gentsch J, Ando T, Grohmann G, Monroe SS, Jiang X, et al. Application of PCR to detect Norwalk virus in fecal specimens from outbreaks of gastroenteritis. J Clin Microbiol 1994;32:642-8.
69. Green J, Norcott JP, Lewis D, Arnold C, Brown DWG. Norwalk-like viruses: demonstration of genomic diversity by polymerase chain reaction. J Clin Microbiol 1993;31:3007-12.
70. Wang J, Jiang X, Madore HP, Gray J, Desselberge U, Ando T, et al. Sequence diversity of small, round-structured viruses in the Norwalk virus group. J Virol 1994;68:5982-90.
71. Ando T, Monroe SS, Gentsch JR, Jin Q, Lewis DC, Glass RL. Detection and differentiation of antigenically distinct small round-structured viruses (Norwalk-like viruses) by reverse transcription-PCR and southern hybridization. J Clin Microbiol 1995;33:64-71.
72. Centers for Disease Control and Prevention. Addressing emerging infectious disease threats: a prevention strategy for the United States. Atlanta (GA): U.S. Department of Health and Human Services, U.S. Public Health Service; 1996.

## References

[PDF_00957] Abbaszadegan M., Gerba C. P., Rose J. B. (1991). Detection of Giardia cysts with a cDNA probe and applications to water samples.. Appl Environ Microbiol.

[PDF_01010] Ando T., Monroe S. S., Gentsch J. R., Jin Q., Lewis D. C., Glass R. I. (1995). Detection and differentiation of antigenically distinct small round-structured viruses (Norwalk-like viruses) by reverse transcription-PCR and southern hybridization.. J Clin Microbiol.

[PDF_00884] Atmar R. L., Metcalf T. G., Neill F. H., Estes M. K. (1993). Detection of enteric viruses in oysters by using the polymerase chain reaction.. Appl Environ Microbiol.

[PDF_00887] Atmar R. L., Neill F. H., Romalde J. L., Le Guyader F., Woodley C. M., Metcalf T. G., Estes M. K. (1995). Detection of Norwalk virus and hepatitis A virus in shellfish tissues with the PCR.. Appl Environ Microbiol.

[PDF_00833] Bean N. H., Goulding J. S., Lao C., Angulo F. J. (1996). Surveillance for foodborne-disease outbreaks--United States, 1988-1992.. MMWR CDC Surveill Summ.

[PDF_00830] Bean N. H., Griffin P. M., Goulding J. S., Ivey C. B. (1990). Foodborne disease outbreaks, 5-year summary, 1983-1987.. MMWR CDC Surveill Summ.

[PDF_00945] Campbell A. T., Robertson L. J., Smith H. V. (1992). Viability of Cryptosporidium parvum oocysts: correlation of in vitro excystation with inclusion or exclusion of fluorogenic vital dyes.. Appl Environ Microbiol.

[PDF_00854] Campbell P. N., Current W. L. (1983). Demonstration of serum antibodies to Cryptosporidium sp. in normal and immunodeficient humans with confirmed infections.. J Clin Microbiol.

[PDF_00810] Cannon R. O., Poliner J. R., Hirschhorn R. B., Rodeheaver D. C., Silverman P. R., Brown E. A., Talbot G. H., Stine S. E., Monroe S. S., Dennis D. T. (1991). A multistate outbreak of Norwalk virus gastroenteritis associated with consumption of commercial ice.. J Infect Dis.

[PDF_00805] Centers for Disease Control and Prevention (CDC) (1993). Multistate outbreak of viral gastroenteritis related to consumption of oysters--Louisiana, Maryland, Mississippi and North Carolina, 1993.. MMWR Morb Mortal Wkly Rep.

[PDF_00801] Centers for Disease Control and Prevention (CDC) (1994). Viral gastroenteritis associated with consumption of raw oysters--Florida, 1993.. MMWR Morb Mortal Wkly Rep.

[PDF_00919] Chung H., Jaykus L. A., Sobsey M. D. (1996). Detection of human enteric viruses in oysters by in vivo and in vitro amplification of nucleic acids.. Appl Environ Microbiol.

[PDF_00907] Deng M. Y., Day S. P., Cliver D. O. (1994). Detection of hepatitis A virus in environmental samples by antigen-capture PCR.. Appl Environ Microbiol.

[PDF_00792] Desenclos J. C., Klontz K. C., Wilder M. H., Nainan O. V., Margolis H. S., Gunn R. A. (1991). A multistate outbreak of hepatitis A caused by the consumption of raw oysters.. Am J Public Health.

[PDF_00728] DuPont H. L., Chappell C. L., Sterling C. R., Okhuysen P. C., Rose J. B., Jakubowski W. (1995). The infectivity of Cryptosporidium parvum in healthy volunteers.. N Engl J Med.

[PDF_00891] Goswami B. B., Koch W. H., Cebula T. A. (1993). Detection of hepatitis A virus in Mercenaria mercenaria by coupled reverse transcription and polymerase chain reaction.. Appl Environ Microbiol.

[PDF_00903] Gouvea V., Santos N., Timenetsky M. do C., Estes M. K. (1994). Identification of Norwalk virus in artificially seeded shellfish and selected foods.. J Virol Methods.

[PDF_01002] Green J., Norcott J. P., Lewis D., Arnold C., Brown D. W. (1993). Norwalk-like viruses: demonstration of genomic diversity by polymerase chain reaction.. J Clin Microbiol.

[PDF_00732] Haas C. N. (1983). Estimation of risk due to low doses of microorganisms: a comparison of alternative methodologies.. Am J Epidemiol.

[PDF_00839] Hayes E. B., Matte T. D., O'Brien T. R., McKinley T. W., Logsdon G. S., Rose J. B., Ungar B. L., Word D. M., Pinsky P. F., Cummings M. L. (1989). Large community outbreak of cryptosporidiosis due to contamination of a filtered public water supply.. N Engl J Med.

[PDF_00749] Hedberg C. W., Osterholm M. T. (1993). Outbreaks of food-borne and waterborne viral gastroenteritis.. Clin Microbiol Rev.

[PDF_00780] Heun E. M., Vogt R. L., Hudson P. J., Parren S., Gary G. W. (1987). Risk factors for secondary transmission in households after a common-source outbreak of Norwalk gastroenteritis.. Am J Epidemiol.

[PDF_00910] Jaykus L. A., De Leon R., Sobsey M. D. (1996). A virion concentration method for detection of human enteric viruses in oysters by PCR and oligoprobe hybridization.. Appl Environ Microbiol.

[PDF_00745] Jenkins S., Horman J. T., Israel E., Cukor G., Blacklow N. R. (1985). An outbreak of Norwalk-related gastroenteritis at a boys' camp.. Am J Dis Child.

[PDF_00755] Kaplan J. E., Feldman R., Campbell D. S., Lookabaugh C., Gary G. W. (1982). The frequency of a Norwalk-like pattern of illness in outbreaks of acute gastroenteritis.. Am J Public Health.

[PDF_00759] Kaplan J. E., Gary G. W., Baron R. C., Singh N., Schonberger L. B., Feldman R., Greenberg H. B. (1982). Epidemiology of Norwalk gastroenteritis and the role of Norwalk virus in outbreaks of acute nonbacterial gastroenteritis.. Ann Intern Med.

[PDF_00880] Lambden P. R., Caul E. O., Ashley C. R., Clarke I. N. (1993). Sequence and genome organization of a human small round-structured (Norwalk-like) virus.. Science.

[PDF_00824] LeChevallier M. W., Norton W. D., Lee R. G. (1991). Giardia and Cryptosporidium spp. in filtered drinking water supplies.. Appl Environ Microbiol.

[PDF_00929] LeChevallier M. W., Norton W. D., Siegel J. E., Abbaszadegan M. (1995). Evaluation of the immunofluorescence procedure for detection of Giardia cysts and Cryptosporidium oocysts in water.. Appl Environ Microbiol.

[PDF_00895] Lees D. N., Henshilwood K., Doré W. J. (1994). Development of a method for detection of enteroviruses in shellfish by PCR with poliovirus as a model.. Appl Environ Microbiol.

[PDF_00899] Lees D. N., Henshilwood K., Green J., Gallimore C. I., Brown D. W. (1995). Detection of small round structured viruses in shellfish by reverse transcription-PCR.. Appl Environ Microbiol.

[PDF_00843] Mac Kenzie W. R., Hoxie N. J., Proctor M. E., Gradus M. S., Blair K. A., Peterson D. E., Kazmierczak J. J., Addiss D. G., Fox K. R., Rose J. B. (1994). A massive outbreak in Milwaukee of cryptosporidium infection transmitted through the public water supply.. N Engl J Med.

[PDF_00973] Mahbubani M. H., Bej A. K., Perlin M. H., Schaefer F. W., Jakubowski W., Atlas R. M. (1992). Differentiation of Giardia duodenalis from other Giardia spp. by using polymerase chain reaction and gene probes.. J Clin Microbiol.

[PDF_00998] Moe C. L., Gentsch J., Ando T., Grohmann G., Monroe S. S., Jiang X., Wang J., Estes M. K., Seto Y., Humphrey C. (1994). Application of PCR to detect Norwalk virus in fecal specimens from outbreaks of gastroenteritis.. J Clin Microbiol.

[PDF_00933] Nieminski E. C., Schaefer F. W., Ongerth J. E. (1995). Comparison of two methods for detection of Giardia cysts and Cryptosporidium oocysts in water.. Appl Environ Microbiol.

[PDF_00836] Ortega Y. R., Sterling C. R., Gilman R. H., Cama V. A., Díaz F. (1993). Cyclospora species--a new protozoan pathogen of humans.. N Engl J Med.

[PDF_00940] Rodgers M. R., Flanigan D. J., Jakubowski W. (1995). Identification of algae which interfere with the detection of Giardia cysts and Cryptosporidium oocysts and a method for alleviating this interference.. Appl Environ Microbiol.

[PDF_00988] Sauch J. F., Flanigan D., Galvin M. L., Berman D., Jakubowski W. (1991). Propidium iodide as an indicator of Giardia cyst viability.. Appl Environ Microbiol.

[PDF_00992] Schupp D. G., Erlandsen S. L. (1987). A new method to determine Giardia cyst viability: correlation of fluorescein diacetate and propidium iodide staining with animal infectivity.. Appl Environ Microbiol.

[PDF_00870] Sobsey M. D., Carrick R. J., Jensen H. R. (1978). Improved methods for detecting enteric viruses in oysters.. Appl Environ Microbiol.

[PDF_00858] Ungar B. L., Soave R., Fayer R., Nash T. E. (1986). Enzyme immunoassay detection of immunoglobulin M and G antibodies to Cryptosporidium in immunocompetent and immunocompromised persons.. J Infect Dis.

[PDF_00937] Vesey G., Slade J. S., Byrne M., Shepherd K., Fricker C. R. (1993). A new method for the concentration of Cryptosporidium oocysts from water.. J Appl Bacteriol.

[PDF_01006] Wang J., Jiang X., Madore H. P., Gray J., Desselberger U., Ando T., Seto Y., Oishi I., Lew J. F., Green K. Y. (1994). Sequence diversity of small, round-structured viruses in the Norwalk virus group.. J Virol.

[PDF_00815] Warner R. D., Carr R. W., McCleskey F. K., Johnson P. C., Elmer L. M., Davison V. E. (1991). A large nontypical outbreak of Norwalk virus. Gastroenteritis associated with exposing celery to nonpotable water and with Citrobacter freundii.. Arch Intern Med.

[PDF_00978] Webster K. A., Pow J. D., Giles M., Catchpole J., Woodward M. J. (1993). Detection of Cryptosporidium parvum using a specific polymerase chain reaction.. Vet Parasitol.

[PDF_00784] White K. E., Osterholm M. T., Mariotti J. A., Korlath J. A., Lawrence D. H., Ristinen T. L., Greenberg H. B. (1986). A foodborne outbreak of Norwalk virus gastroenteritis. Evidence for post-recovery transmission.. Am J Epidemiol.

[PDF_00877] Xi J. N., Graham D. Y., Wang K. N., Estes M. K. (1990). Norwalk virus genome cloning and characterization.. Science.

